# Novel TaqMan PCR Assay for the Quantification of *Paenibacillus larvae* Spores in Bee-Related Samples

**DOI:** 10.3390/insects12111034

**Published:** 2021-11-17

**Authors:** Darja Kušar, Bojan Papić, Urška Zajc, Irena Zdovc, Majda Golob, Lucija Žvokelj, Tanja Knific, Jana Avberšek, Matjaž Ocepek, Metka Pislak Ocepek

**Affiliations:** 1 Institute of Microbiology and Parasitology, Veterinary Faculty, University of Ljubljana, Gerbičeva 60, 1000 Ljubljana, Slovenia; bojan.papic@vf.uni-lj.si (B.P.); urska.zajc@vf.uni-lj.si (U.Z.); irena.zdovc@vf.uni-lj.si (I.Z.); majda.golob@vf.uni-lj.si (M.G.); jana.avbersek@vf.uni-lj.si (J.A.); matjaz.ocepek@vf.uni-lj.si (M.O.); 2 Institute of Pathology, Wild Animals, Fish and Bees, Veterinary Faculty, University of Ljubljana, Gerbičeva 60, 1000 Ljubljana, Slovenia; lucija.zvokelj@vf.uni-lj.si (L.Ž.); metka.pislakocepek@vf.uni-lj.si (M.P.O.); 3 Institute of Food Safety, Feed and Environment, Veterinary Faculty, University of Ljubljana, Gerbičeva 60, 1000 Ljubljana, Slovenia; tanja.knific@vf.uni-lj.si

**Keywords:** American foulbrood (AFB), *Paenibacillus larvae*, real-time or quantitative PCR (qPCR), digital PCR (dPCR), plate counting, spore germination rate, honey, hive debris

## Abstract

**Simple Summary:**

American foulbrood (AFB) is the most severe bacterial disease of honeybees, caused by *Paenibacillus larvae*. Larvae become infected by ingesting food contaminated with *P. larvae* spores, which are extremely resistant and can remain infectious for decades. Burning affected colonies is widely used to prevent further spread of the disease. The presence of *P. larvae* spores in bee-related samples is associated with an increased risk of developing clinical symptoms, and spore counts can be used for early detection of at-risk colonies, which should then undergo thorough clinical examination. Because quantification of *P. larvae* spores by plate counting is time-consuming and unreliable, due to poor and inconsistent germination, molecular quantification is more suitable. To overcome the limitations of available quantification methods, we developed a quantitative PCR (qPCR) assay for reliable quantification of *P. larvae* that also performs well at low spore counts. The assay was validated for honey and hive debris samples but can be extended to other sample types. Spore counts in AFB-positive colonies were significantly higher than those in asymptomatic colonies, both for honey and hive debris samples. By comparing plate and qPCR counts, the germination rate of *P. larvae* spores was found to be low and inconsistent.

**Abstract:**

*Paenibacillus larvae* is the causative agent of American foulbrood (AFB), a devastating disease of honeybees. *P. larvae* spore counts in bee-related samples correlate with the presence of AFB symptoms and may, therefore, be used to identify at-risk colonies. Here, we constructed a TaqMan-based real-time PCR (qPCR) assay targeting a single-copy chromosomal metalloproteinase gene for reliable quantification of *P. larvae*. The assay was calibrated using digital PCR (dPCR) to allow absolute quantification of *P. larvae* spores in honey and hive debris samples. The limits of detection and quantification were 8 and 58 spores/g for honey and 188 and 707 spores/mL for hive debris, respectively. To assess the association between AFB clinical symptoms and spore counts, we quantified spores in honey and hive debris samples originating from honeybee colonies with known severity of clinical symptoms. Spore counts in AFB-positive colonies were significantly higher than those in asymptomatic colonies but did not differ significantly with regard to the severity of clinical symptoms. For honey, the average spore germination rate was 0.52% (range = 0.04–6.05%), indicating poor and inconsistent in vitro germination. The newly developed qPCR assay allows reliable detection and quantification of *P. larvae* in honey and hive debris samples but can also be extended to other sample types.

## 1. Introduction

Honeybees (*Apis mellifera*) play an essential role in pollination and biodiversity conservation [1]. Several stress factors affect bee health on national and global scales, leading to significant reductions of their populations [2,3]. Biotic factors include pathogens from both prokaryotic or eukaryotic taxa and pests causing various diseases of bees. On the other hand, bee health is endangered by abiotic stressors, including unfavorable weather conditions, habitat or diet changes, introduction of invasive species, intoxication of beehives with acaricides, and treatment of crops with insecticides.

American foulbrood (AFB) is one of the most widespread and most severe diseases of honeybees and is caused by the spore-forming Gram-positive bacterium *Paenibacillus larvae* [4]. Honeybee larvae become infected by ingesting food contaminated with *P. larvae* spores, which are highly resistant to environmental factors and can remain infectious for several decades [5]. Clinical onset of AFB depends on the virulence and spore count of *P. larvae* in the honeybee colony as well as many intrinsic (genetic) and extrinsic (environmental) factors [6,7]. In Slovenia, the disease is diagnosed when characteristic clinical symptoms are identified in the honeybee colony and the causative agent is confirmed by laboratory examination [8]. Due to its severity, AFB is a statutory notifiable disease in the European Union (Council Directive 92/65/EEC, 1992) and is regulated by national legislation in many countries worldwide, including Slovenia [8]. Elimination of the infected honeybee colonies and contaminated equipment is required, and trade restrictions are enforced in the outbreak area, resulting in significant economic losses to the beekeeping industry. Early detection of the disease is the most effective way to prevent its spread.

Apparently healthy honeybee colonies can harbor *P. larvae* spores [9,10,11]. Spore counts in honey, hive debris, and adult bees correlate with the severity of symptoms in the clinical stage of AFB and may be useful in identifying colonies in the pre-clinical stage of the disease [10,11,12,13,14,15,16,17]. Thus, quantification of *P. larvae* spores offers a promising prognostic tool for early detection of at-risk apiaries based on increased spore counts in the colonies, which should then undergo thorough clinical examination. For this purpose, a method for reliable quantification of *P. larvae* spores in different samples is needed.

Honey and (winter) hive debris samples provide non-invasive and easily accessible material for *P. larvae* surveillance. Moreover, they may reflect a long-term accumulation of *P. larvae* spores in the hive, whereas spore counts in adult bees mostly reflect the current disease status of the colony [12,15]. Previous studies that used culture-based methods for the detection and quantification of *P. larvae* showed a limited ability of honey to reliably identify diseased colonies, since spores may remain undetected even in symptomatic colonies [9,10]. On the contrary, spore counts in accumulated winter hive debris have shown promise in reflecting disease status, since hive debris allowed identification of higher numbers of *P. larvae*-positive colonies compared with adult bees, regardless of disease status [15], and was also a better predictor of the onset of AFB [11]. In Slovenia, it is common practice to collect honey samples from colonies in areas with an increased risk of AFB to monitor *P. larvae* spore counts by cultivation, whereas hive debris is usually not present during clinical examinations of apiaries in summer. Sampling of adult bees for spore counts has not yet been introduced into our laboratory as of the time of this study.

Two main approaches for the quantification of *P. larvae* spores in bee-related samples are culture- and PCR-based methods. To date, the detection and quantification of *P. larvae* in honey and hive debris have mostly relied on culture-based methods [9,11,17,18]. However, these suffer from certain limitations. First, germination of *P. larvae* spores is markedly affected by the sample pretreatment procedure, *P. larvae* genotype, type of honey, and the choice of culture media [19,20,21,22]. Moreover, the method is time-consuming and requires species confirmation [23]. Thus, alternative and state-of-the-art methods for reliable detection and quantification of *P. larvae* are needed to reassess the relationship between spore counts in bee-related samples and the disease status of the corresponding honeybee colonies.

Quantitative real-time PCR (qPCR) offers a time- and cost-effective alternative for quantifying *P. larvae* spores and overcomes the limitations of culture-based methods. Although several qPCR assays have been developed for the detection and characterization of *P. larvae* in different sample types [21,23,24,25,26,27,28], only two assays have been optimized for the quantification of *P. larvae* spores in honey and/or hive debris samples [23,26]. Both target the 16S rRNA gene and use SYBR technology, which suffer from important limitations. Moreover, in these two studies, the standard curve for qPCR quantification was based on plate counts [23] or flow cytometry [26]. The latter requires a clean culture (suspension of vegetative cells or spores) and is, therefore, unsuitable for direct quantification of *P. larvae* in complex sample types (e.g., bee-related samples).

Contrary to flow cytometry, digital PCR (dPCR) allows direct quantification of the target in complex sample types and uses the same DNA template as well as amplification and detection chemistry as qPCR. dPCR allows absolute quantification without the need for a standard curve [29] but, to our knowledge, has not yet been used for *P. larvae*. Here, we developed a novel TaqMan-based qPCR assay for the quantification of *P. larvae* which was validated for honey and hive debris samples and calibrated using dPCR to allow absolute quantification. For honey samples, spore counts derived from qPCR were compared with those derived from plate counting, and the correlation between spore counts and the intensity of AFB clinical symptoms in the corresponding honeybee colonies was assessed. The latter was also investigated for hive debris samples.

## 2. Materials and Methods

### 2.1. Samples

Sampling of honey and hive debris was performed in 2019–2020. In most cases, only one sample type (honey or hive debris) per apiary was obtained. Both sample types were collected in sterile containers from individual honeybee colonies. Honey samples were collected from the honeycomb cells near the brood within the scope of veterinary clinical examinations of colonies upon clinical suspicion of AFB; they originated from AFB-positive (*n* = 41) or asymptomatic (*n* = 26) colonies positioned in AFB-positive apiaries (Table S1). To collect hive debris, sampling boards were placed at the bottom of hives in apiaries with different AFB status. One month after placing the boards, a control examination of colonies was performed. Hive debris samples were collected from newly clinically affected (i.e., AFB-positive or symptomatic) colonies (*n* = 17) and asymptomatic colonies positioned in AFB-positive apiaries (*n* = 52). Hive debris was also collected from colonies positioned in asymptomatic apiaries with a history of AFB and/or located within the active AFB zone (*n* = 25) or apiaries with a complete absence of AFB (*n* = 13) (Table S1).

#### 2.1.1. Honey

For the quantification of *P. larvae* spores by plate counting and qPCR, 67 honey samples were collected (Table S1). The colonies had AFB clinical symptoms of various severities (0–4; Table S2) and originated from 18 apiaries (1–18); more than one honeybee colony per apiary was sampled for 13 apiaries (Table S1). Honey samples were heated to 45–50 °C, and 50 g was poured into sterile centrifuge containers, there supplemented with 150 mL of dH_2_O (50 °C), and mixed to a homogeneous suspension. Samples were centrifuged at 4000× *g* for 30 min, and the supernatant was discarded, leaving ~1 mL to resuspend the pellet. The resulting suspension was supplemented with sterile 0.9% NaCl (saline) to a final volume of 10 mL and incubated at 80 °C for 10 min to kill the vegetative cells (suspension H1).

For quantification by plate counting, the prepared suspension was diluted by a factor of 10 once more (1 mL of suspension H1 supplemented with 9 mL of sterile saline; suspension H2). A total of 500 µL of suspensions H1 and H2 was inoculated onto Brain heart infusion (BHI; Oxoid, Basingstoke, UK) agar plates supplemented with 5% sheep blood, 1 mg/L thiamine (Fargon Hellas, Trikala, Greece), and 30 mg/L nalidixic acid (MilliporeSigma by Merck, Burlington, MA, USA) using Drigalski spatulas. Plates were incubated at 37 °C for seven days. Presumptive *P. larvae* colonies were counted, and one colony per plate was confirmed by matrix-assisted laser desorption/ionization time-of-flight (MALDI-TOF) mass spectrometry (Microflex LT system; Bruker Daltonics, Leipzig, Germany) according to the manufacturer’s instructions.

For qPCR quantification, 1 mL of suspension H1 was used for DNA extraction, corresponding to 5 g of honey sample (1/10 of the initial sample). For qPCR validation, a single naturally contaminated honey sample (positive by bacteriological examination) was used; three 1 mL aliquots of suspension H1 were collected to represent three biological replicates used for DNA extraction. A honey sample determined as negative by bacteriological examination and showing no qPCR amplification curve in the preliminary analysis was used as a negative template control (NTC).

#### 2.1.2. Hive Debris

*P. larvae* spores in the hive debris samples were quantified by qPCR; plate counting was not performed, because we had observed poor spore germination for honey samples in our previous laboratory examinations. A total of 107 hive debris samples were quantified (Table S1); pooled samples were obtained from three apiaries without current AFB symptoms in any of the colonies (20, 29, and 30 in Table S1). The colonies had varying severities of AFB clinical symptoms (0–2; Table S2) and originated from 16 apiaries (17–32); for 11 apiaries, more than one honeybee colony per apiary was sampled for individual (non-pooled) quantification (Table S1). Volume (mL) was selected as the basic metric unit for hive debris samples, due to the variation in moisture content. A total of 5 mL of the collected hive debris was transferred to a 50 mL centrifuge tube and supplemented with sterile saline to a final volume of 50 mL. The suspension was vigorously mixed (KS 4000 i control; IKA, Staufen, Germany) at 160 rpm for 2 h and homogenized (MiniMix; Interscience, Saint Nom, France) at the highest speed for 1.5 min (suspension D1).

For qPCR quantification, 1 mL of suspension D1 was used for DNA extraction, corresponding to 0.1 mL of hive debris sample (1/50 of the initial sample). A single naturally contaminated hive debris sample originating from an AFB-positive colony was used for qPCR validation; three 1 mL aliquots of suspension D1 were collected to represent three biological replicates used for DNA extraction. One of the negative hive debris samples originating from an apiary with a complete absence of AFB was used as a NTC.

### 2.2. DNA Extraction

The extraction of DNA from honey and hive debris samples was performed using a commercial kit (DNA isolation from complex samples, Institute of Metagenomics and Microbial Technologies, Ljubljana, Slovenia; https://www.immt.eu, accessed on 12 November 2021) according to the manufacturer’s instructions. The protocol included bead-beating combined with enzymatic and heat-induced lysis between mechanical shearing steps. Briefly, 1 mL of suspensions H1 and D1 was centrifuged at 10,000× *g* for 5 min in 2 mL screw-cap tubes containing 150 mg of glass beads with diameter ≤106 µm (MilliporeSigma by Merck, Burlington, MA, USA). The supernatants were discarded, and lysis buffer (392 µL of destroy buffer D) supplemented with 8 µL of proteinase K (20 mg/mL; MilliporeSigma by Merck, Burlington, MA, USA) was added to the pellets. Twice, samples were subjected to bead-beating (45 s at 6400 rpm) using a tissue homogenizer (MagNA Lyser Instrument; Roche, Basel, Switzerland) and incubation at 56 °C for 15 min. After the third beat-beating, samples were incubated at 100 °C for 10 min and cooled in the refrigerator for 3 min. After centrifugation at 10,000× *g* for 5 min, supernatants were mixed with three times the volume of binding buffer B and loaded onto spin columns for centrifugation at 16,000× *g* for 1 min. After rinsing two times with wash buffer W, DNA was eluted from the spin columns with elution buffer E. For DNA extraction, 1 mL of the prepared sample suspensions (Section 2.1.1 and Section 2.1.2) was used, and DNA was eluted to a final volume of 100 µL; these volumes, together with DNA dilutions and DNA-to-PCR-mixture volume ratios, were considered when calculating *P. larvae* spore numbers per sample unit (g for honey and mL for hive debris) from qPCR (Cq values) and dPCR (copies of target per µL of the extracted DNA).

### 2.3. Design of a Quantitative P. larvae TaqMan Assay

We selected the chromosomal metalloproteinase (MP) gene (NCBI genome accession number CP020557.1, locus tag B7C51_23310; [30]) as the target gene, because it is present in a single copy in all *P. larvae* genomes and is highly conserved (≥99.7% identity and 100% coverage) among the currently established *P. larvae* ERIC types [4,31]. Primers (MPF, MPR) and probe (MPP) for its amplification were constructed with the IDT RealTime qPCR Assay Entry tool (https://eu.idtdna.com/scitools/Applications/RealTimePCR/ accessed on 14 March 2019) and analyzed, using the IDT OligoAnalyzer tool (https://eu.idtdna.com/calc/analyzer accessed on 14 March 2019), by applying the qPCR parameter sets. The TaqMan probe was labeled with the reporter 6-FAM at the 5′-end (56-FAM), with a double quencher, ZEN, as an internal quencher, and Iowa Black FQ at the 3′-end (3IABkFQ; Integrated DNA Technologies [IDT], Coralville, IA, USA). The constructed 5′–3′ sequences were GGT AAC TAT TCT GGC AGG AGC for the forward primer (MPF), AAG TTC ACG GTT AGG GTC TTC for the reverse primer (MPR), and [56-FAM] TTG GTA GGA [ZEN] ACG TCA TTG TCC GCA [3IABkFQ] for the probe (MPP).

### 2.4. In Silico and In Vitro Inclusivity and Exclusivity of P. larvae TaqMan Assay

In silico inclusivity was assessed based on nine *P. larvae* complete genomes available in the NCBI Genomes Database (Table S3; accessed on 30 July 2021). In addition, inclusivity was tested based on 40 field *P. larvae* isolates from Slovenia of different ERIC types with available whole-genome sequencing (WGS) data (Table S3). Metalloproteinase gene sequences were identified using BLASTn by applying an identity of ≥90% and a coverage of ≥70% as cut-off values. Gene alignments were performed and visualized using the Geneious aligner implemented in Geneious v11.1.5 (Biomatters, Auckland, New Zealand).

To assess in silico exclusivity, the five most closely related *Paenibacillus* species with available complete or draft genome data were identified based on the highest identity of the 16S rRNA gene using the EzBioCloud identification service [32] (Table S3). BLASTn was used to identify the homologs of the metalloproteinase gene by applying a cut-off of ≥90% identity and ≥70% coverage.

A total of 23 field *P. larvae* isolates of different ERIC types and five *P. larvae* ERIC reference strains were used to assess in vitro inclusivity (Table S4). Two related species (*Bacillus pumilus* and *Paenibacillus alvei*) and five common honeybee pathogens (*Chritidia mellificae*, *Lotmaria passim*, *Melissococcus plutonius*, *Nosema apis*, and *Nosema ceranae*) were used to assess in vitro qPCR exclusivity (Table S4).

### 2.5. qPCR and dPCR Conditions

The qPCR reaction mix contained 10 μL of 2× master mix (Maxima Probe/ROX qPCR MasterMix; Thermo Fisher Scientific, Waltham, MA, USA), 0.12 μL of passive reference dye ROX (diluted 1:10), 300 nM of both primers and 200 nM of probe, 5 μL of template DNA (undiluted or from a dilution series prepared for method validation; Section 2.6), and PCR-grade water to a final volume of 20 μL. Amplification and detection were performed on the 7500 Fast Real-Time PCR System (Applied Biosystems by Thermo Fisher Scientific, Foster City, CA, USA). The following amplification protocol was used: 50 °C for 2 min, 95 °C for 10 min, and 45 cycles of 95 °C for 15 s and 60 °C for 1 min. Results were expressed in quantification cycle (Cq) values. After validation (Section 2.6), the threshold line was set at 0.1 for all samples. Positive control, NTC, and water no template control (WNTC) were included in each run.

For dPCR, the same TaqMan assay was employed as for qPCR. The reaction mix contained 7.5 μL of 2× master mix (QuantStudio 3D Digital PCR Master Mix v2; Applied Biosystems by Thermo Fisher Scientific, Foster City, CA, USA), 300 nM of both primers and 200 nM of probe, 3 μL of template DNA (appropriately diluted to allow dPCR quantification or selected from a dilution series prepared for qPCR validation), and PCR-grade water to a final volume of 15 μL. The prepared dPCR reactions were loaded onto QuantStudio 3D Digital PCR 20K Chips v2 (consisting of 20,000 reaction wells per chip), and amplification was performed on a ProFlex 2× Flat PCR System (Applied Biosystems by Thermo Fisher Scientific, Foster City, CA, USA) according to the manufacturer’s instructions. The following amplification protocol was employed: 96 °C for 10 min, 39 cycles of 60 °C for 2 min and 98 °C for 30 s, and 60 °C for 2 min. After chip imaging and initial analysis with the QuantStudio 3D Digital PCR Instrument, results were analyzed using QuantStudio 3D AnalysisSuite v3.1.6 Cloud Software; after validation (Section 2.6), the quality and fluorescence (FAM) threshold values were set at 0.6 and 2000, respectively. Results were expressed in copies/μL (i.e., number of single-copy metalloproteinase gene targets, corresponding to the number of *P. larvae* spores per μL of the extracted DNA), considering the DNA dilution rate and its volume in the PCR mix. The precision of dPCR, which refers to the ability to discriminate between two measurements with a certain confidence (the lower the precision, the narrower the confidence interval), was calculated by the software. Positive control, NTC, and WNTC were included in each run.

### 2.6. Validation of qPCR and Its Calibration Using dPCR

For the quantification of *P. larvae* spores, linear regression of a standard curve was performed to validate qPCR. Three 1 mL aliquots of the suspensions prepared from a naturally contaminated honey and hive debris sample (see Section 2.1.1 and Section 2.1.2) represented three biological replicates for DNA extraction, all of which were analyzed in three technical replicates, resulting in nine qPCR results per dilution; the extracted DNA was diluted in a 5-fold dilution series (for honey, the first two dilutions were 10-fold, followed by a 5-fold dilution series). For the calculation of a linear regression equation, only the data within the linear dynamic range were considered, defined as dilutions with the coefficient of variation (CV) below 33%, since the CV markedly increases below the LOQ [33]. The CV was defined for each dilution as the ratio between the standard deviation of the calculated spore concentration and the average calculated concentration; spore concentrations were calculated from the obtained regression equation. The qPCR amplification efficiency was calculated according to the equation E = 10^−1/slope^ − 1 [33].

Considering the obtained CVs, the limit of quantification (LOQ) and Cq cut-off value were determined both for honey and hive debris. The LOQ was defined from the last serial dilution with CV below 33%, indicating the lower limit of the linear dynamic range. For determination of the Cq cut-off value, the first standard dilution where no amplification was observed in some of the replicates was considered; the highest value was selected from Cq values obtained for this dilution, rounded up to the next half value, and increased by 0.5 to determine the cut-off value [34]. From the LOQ, the limit of detection (LOD) was determined by considering the next dilution in the series, since LOD is 5–10 times lower than LOQ in complex samples [35].

To calibrate qPCR using dPCR, the prepared DNA dilution series was also quantified using dPCR. For dPCR, all three biological replicates were analyzed in one technical replicate. Since dPCR is characterized by a narrower dynamic range [29], only the three most optimal dilutions were considered. The obtained dPCR results were reported as the number of targets per µL of DNA and were used for the calibration of qPCR. For the conversion of Cq values to spores per sample unit, all dilutions of the standard curve from sample preparation to PCR were considered. For field samples, spores per sample unit were calculated from Cq values according to the standard curve equation.

### 2.7. Agreement between qPCR and dPCR

After calibration, the agreement between qPCR and dPCR measurements was assessed using Bland–Altman analysis and Spearman’s correlation. For this purpose, additional honey (*n* = 17) and hive debris (*n* = 24) samples (obtained independently of the samples shown in Table S1) were quantified using qPCR and dPCR, as described in Section 2.5. For honey samples, the correlation between plate and qPCR counts was also assessed.

For dPCR, a single chip per sample was run, and those with 20–10,000 copies/µL of the analyzed DNA were used for comparison. According to the manufacturer’s instructions, the best results are obtained when the concentration of the target sequence per reaction mix is within the optimal range of 200–2000 copies/µL, where precision of at least 10% at a 95% confidence interval can be achieved. Because dPCR measurements were in high agreement with those of qPCR, even outside this dynamic range, the acceptable precision was arbitrarily set at <30%, extending the dynamic range of dPCR to 20–10,000 copies/µL and allowing dPCR/qPCR comparison on a larger number of samples.

All samples with dPCR precision <30% were quantified using qPCR run in triplicate, and the average spore count was considered. For statistical analyses, all spore counts were log-transformed and a *p*-value of <0.05 was considered significant. GraphPad Prism v8.0.2 (GraphPad Software, San Diego, CA, USA) was used to generate Bland–Altman plots and calculate Spearman’s correlation.

### 2.8. Association between Spore Counts and Clinical Symptoms

To assess the association between AFB clinical symptoms and spore counts, a set of 67 honey and 107 hive debris samples originating from honeybee colonies (hives) with known severity of clinical symptoms of AFB were used (Table S1). AFB clinical symptoms were rated on a scale of 0 to 4, with “0” representing no clinical symptoms and “4” representing the highest severity of clinical symptoms (Table S2). Spore counts per sample unit were determined by qPCR (both sample types) and plate counting (honey); Spearman’s coefficient was used to assess the correlation between the two methods. Honey samples with counts above the LOQ by both methods were also included in the calculation of spore germination rate, which was defined as the ratio between plate and qPCR counts (expressed as a percentage). For qPCR, LOQ was determined during validation and calibration of the method (Section 2.6). For plate counting, plates with 15–300 colony forming units (CFUs) were considered suitable for enumeration. For the purpose of statistical analyses, all values below the LOQ of plate counting or qPCR were set at 0.5 LOQ value (Table S1).

A Kruskal–Wallis test, followed by Dunn’s post hoc multiple comparison test, was used to evaluate the differences in spore counts of *P. larvae* with respect to the severity of clinical symptoms of AFB. The Mann–Whitney test was used to compare spore counts in the asymptomatic colonies with different histories of AFB. All of the above tests were performed using GraphPad Prism v8.0.2 (GraphPad Software, San Diego, CA, USA). The univariate logistic regression in R statistical software v4.0.5 [36] was used to investigate whether *P. larvae* spore counts determined by qPCR (honey and hive debris) or plate counting (honey) have the potential to classify colonies as symptomatic (i.e., clinically affected) or asymptomatic (i.e., clinically unaffected). A *p*-value of <0.05 was considered significant.

## 3. Results

### 3.1. In Silico and In Vitro Inclusivity and Exclusivity of P. larvae TaqMan Assay

The assay showed perfect in silico inclusivity with no mismatches observed in the primer/probe binding sites (Figure S1, Table S3). In silico exclusivity was also perfect, since none of the five most closely related *Paenibacillus* species harbored metalloproteinase homologs (Table S3). Moreover, a BLASTn search yielded no hits with identity of ≥90% and coverage of ≥70% in the complete NCBI nr/nt database when the complete metalloproteinase gene was used as a query and the taxon ‘*Paenibacillus larvae*’ was excluded.

After establishing reaction conditions (Section 2.5) and performing validation (Section 2.6), field and reference strains underwent qPCR. None of the tested non-*P. larvae* isolates were qPCR-positive, whereas all *P. larvae* isolates were positive (Table S4); thus, the assay also showed perfect (100%) in vitro inclusivity and exclusivity. Furthermore, the expected qPCR amplicon length (94 bp) was confirmed by separation by QIAxcel capillary electrophoresis (Qiagen, Hilden, Germany) using the QIAxcel DNA High Resolution Kit, QX Alignment Marker 15–1000 bp, QX Size Marker 50–800 bp, and OM500 separation method (data not shown).

### 3.2. Validation of qPCR and Its Calibration Using dPCR

**Figure 1:**
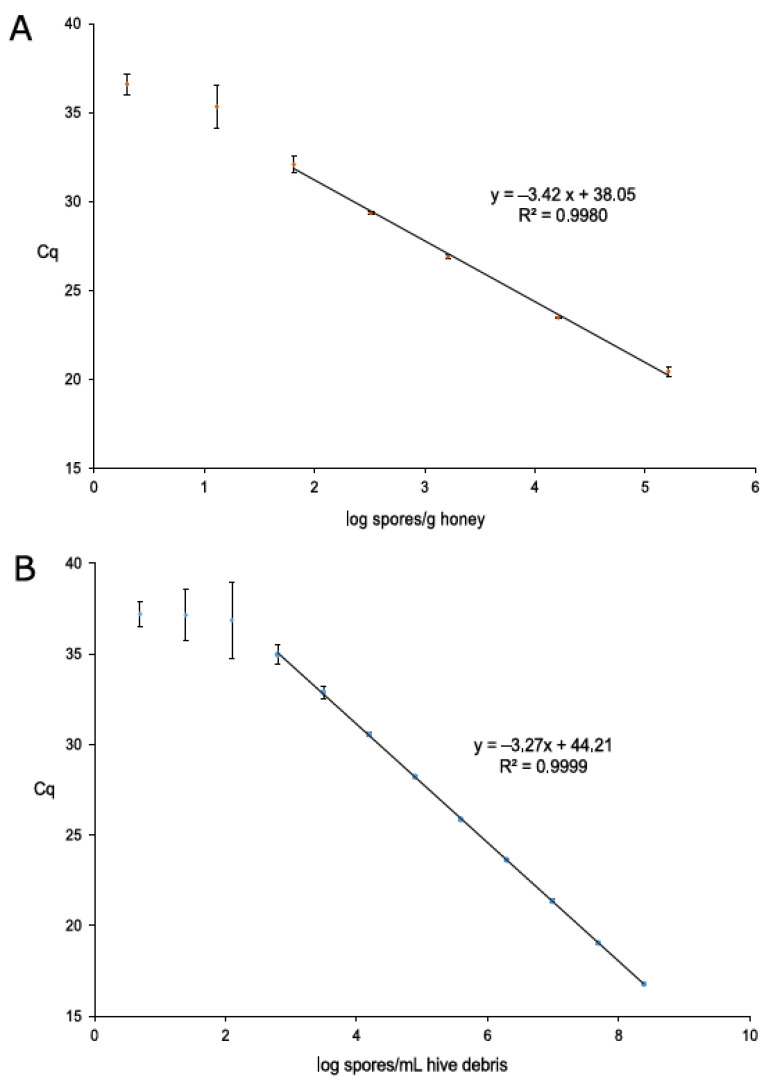
Standard curves for qPCR quantification of *Paenibacillus larvae* in honey (**A**) and hive debris (**B**). Each of the three biological replicates per dilution was measured in three technical replicates, giving a total of nine measurements per dilution. Performance parameters (standard curve equation and R^2^) are given. Dilutions outside the linear range (i.e., dilutions where CV was greater than 33%; two for honey and three for hive debris) are also shown.

Standard curves for honey and hive debris were based on average Cq values obtained from nine replicates of the quantitative serial dilutions plotted against the estimated number of *P. larvae* spores per sample unit (g of honey and mL of hive debris) in logarithmic values; absolute quantification by qPCR was possible after its calibration using dPCR, which reported the absolute number of targets (*P. larvae* spores) per µL of DNA extracted from positive honey and hive debris calibration samples (Table 1). Parameters for both qPCR standard curves were within the bounds of expected performance (Figure 1), with amplification efficiencies of 96.0% for honey and 102.1% for hive debris.

According to the CVs, LOQ values were set at 58 *P. larvae* spores/g honey and 707 *P. larvae* spores/mL hive debris, and LOD values at 8 *P. larvae* spores/g honey and 188 *P. larvae* spores/mL hive debris; from a 5-fold dilution series, the LOD was calculated from the dilution which was the first one higher than the LOQ dilution, generating a LOD approximately five times lower than LOQ. Considering all dilutions during DNA extraction and qPCR mix preparation, LOD values could be translated into 2 and 1 *P. larvae* spores/qPCR reaction for honey and hive debris, respectively, reaching the reported theoretical limit of three target copies per PCR [37]. The Cq cut-off value was set at 38.0 for both sample types.

### 3.3. Agreement between qPCR and dPCR

Based on the results of dPCR, 14 honey and 12 hive debris samples with 20–10,000 copies/µL were selected for comparison of dPCR and qPCR (Table S5). For honey, 9/14 samples showed a precision of <10% (range = 1.9–5.7%) and 5/14 a reasonable precision of <30% (range = 17.6–27.8%). For hive debris, 8/12 samples showed high precision (range = 2.0–14.5%), and 4/12 showed acceptable precision in the range of 24.2–26.8%. For dPCR, the number of spores ranged from 490 to 190,506 per g of honey and 27,512 to 9,756,100 per mL of hive debris. For qPCR, the number of spores ranged from 534 to 315,604 per g of honey and from 18,249 to 8,313,821 per mL of hive debris; the same samples (H5, H12, D3, and D12) were at the extreme ends of the range both by dPCR and qPCR (highlighted in Table S5).

**Figure 2:**
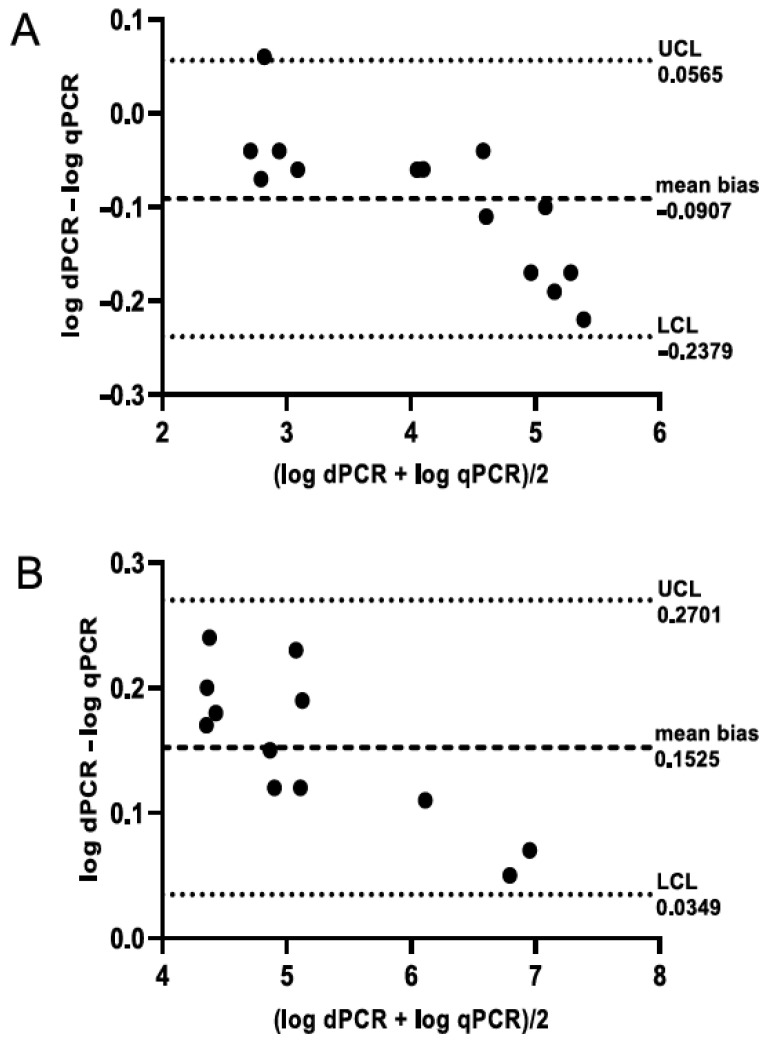
Bland–Altman plot showing the agreement between qPCR and dPCR for honey (**A**) and hive debris (**B**) samples. A total of 14 honey and 12 hive debris samples with spore counts above the limit of quantification by both methods were included in the analysis; see Section 2.7 for details. LCL, lower confidence level; UCL, upper confidence level.

Bland–Altman analysis showed high agreement between qPCR and dPCR, both for honey (Figure 2A) and hive debris (Figure 2B), indicating interchangeability of both methods and successful calibration of qPCR using dPCR. Although it could be noted that qPCR had slightly higher values than dPCR for honey samples and the opposite was true for hive debris (Table S5), the mean bias value was close to zero for both sample types. Furthermore, the correlation between both methods was highly significant (*p* < 0.0001), with Spearman’s *r_s_* = 0.9912 for honey, 0.9492 for hive debris, and 0.9682 for both sample types combined.

### 3.4. Association between Spore Counts and Clinical Symptoms

For honey (*n* = 67) and hive debris (*n* = 107) samples collected from colonies with varying severity of clinical symptoms (honey: 26 samples with level “0”, 14 with “1”, 17 with “2”, nine with “3”, and one with “4”, which was regarded as “3” for the purpose of statistical analyses; hive debris: 96 samples with level “0”, nine with “1” and eight with “2”), spore counts were determined by plate counting (honey) and qPCR (honey and hive debris) (Table S1). For apiaries, we indicated if AFB was active (i.e., clinical symptoms were present in at least one colony per apiary; asymptomatic colonies from such AFB-positive apiaries were also included in the sampling) or completely absent (i.e., the apiary was free from AFB, since it contained no clinically affected colonies, was not located in any of the active AFB zones, and had no history of AFB). As expected, qPCR counts in hive debris samples originating from asymptomatic colonies (clinical symptoms designated as “0”) were significantly higher in apiaries with a history of AFB than in those with full absence of AFB (*p* < 0.0001, Mann–Whitney test); no such association could be assessed for honey samples, since all originated from AFB-positive apiaries. In addition, spore counts in hive debris samples collected from asymptomatic colonies with a history of AFB were significantly lower than in those from affected colonies with clinical symptoms designated as “1” (*p* < 0.0001, Mann–Whitney test).

**Figure 3:**
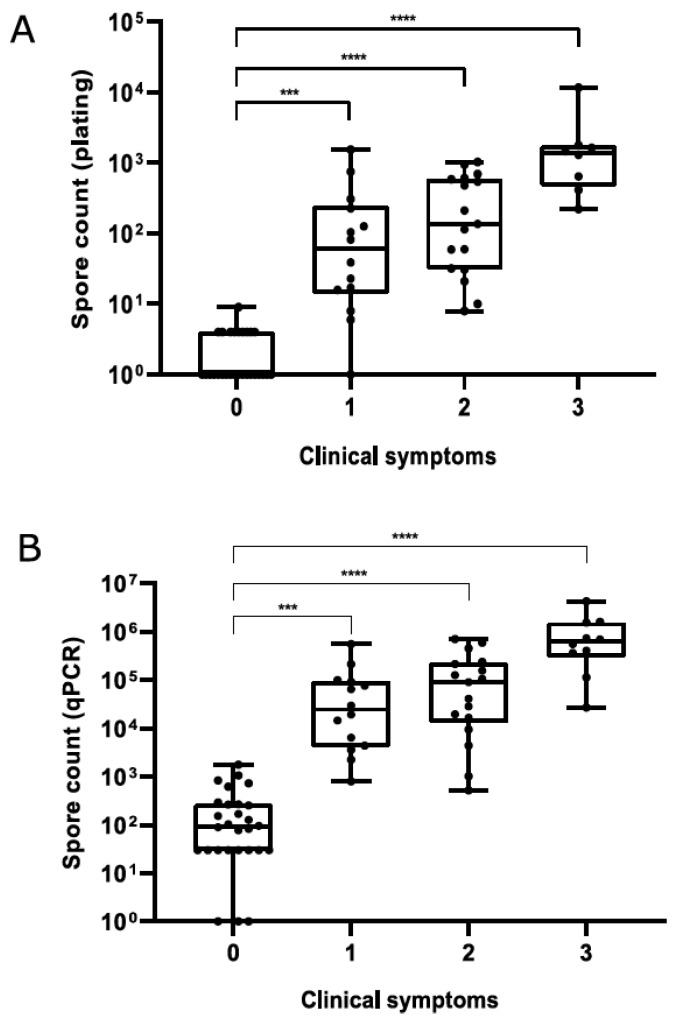
*Paenibacillus larvae* spore counts in honey samples determined by plate counting (**A**) and qPCR (**B**) with respect to the severity of clinical symptoms. Significant differences (Kruskal–Wallis test followed by Dunn’s post hoc test) are marked with asterisks. Legend: ***, *p* ≤ 0.001; ****, *p* ≤ 0.0001.

**Figure 4:**
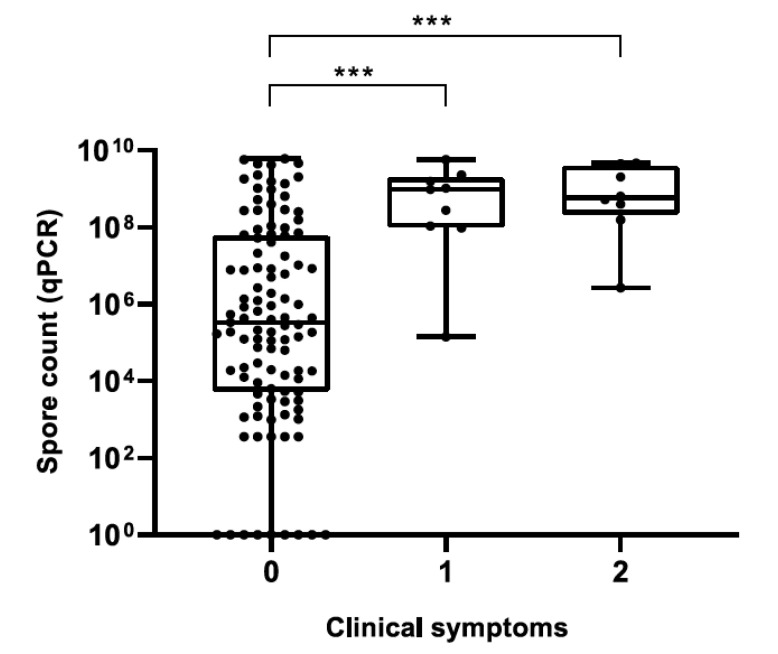
*Paenibacillus larvae* spore counts in hive debris samples determined by qPCR with respect to the severity of clinical Scheme 0. Legend: ***, *p* ≤ 0.001.

For honey samples, a significant positive correlation was observed between plate and qPCR counts (Spearman’s *r_s_* = 0.9185, *p* < 0.0001), although plate counts were always lower than qPCR counts (Table S1). Median spore counts in honey (Figure 3) and hive debris (Figure 4) samples from clinically affected colonies were significantly different (*p* < 0.001) from those in asymptomatic colonies; for honey samples, this was observed for both quantification methods. Although a trend of increased spore counts with increasing severity of clinical symptoms was observed, median spore counts in AFB-positive samples did not differ significantly with respect to the severity of AFB symptoms, regardless of sample type.

**Figure 5:**
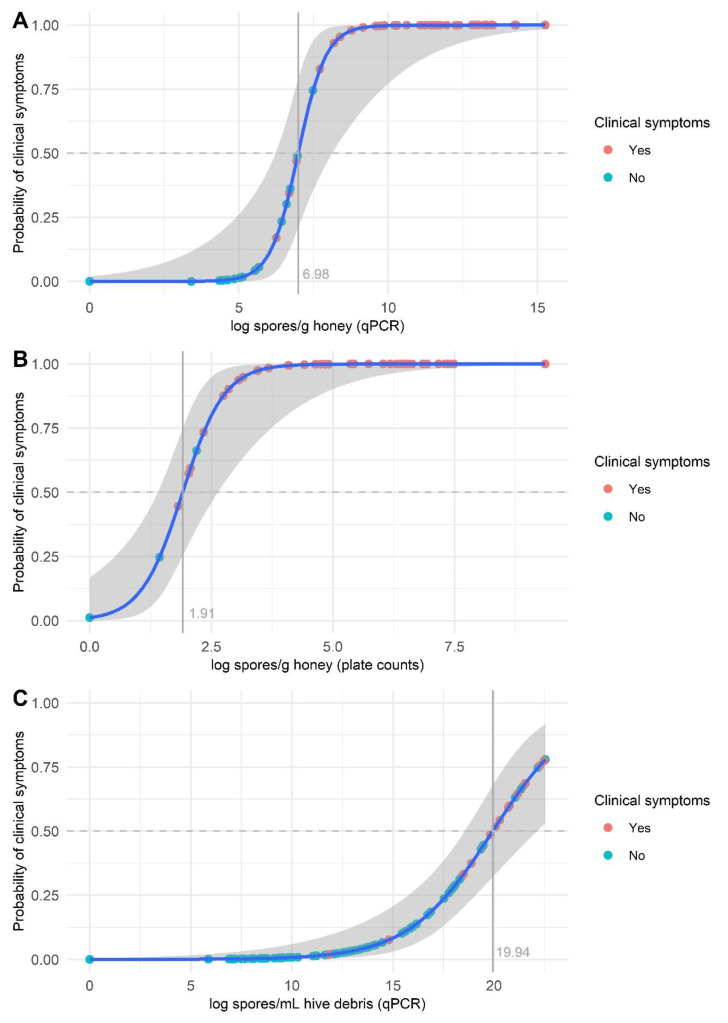
Logistic regression plot (blue line, logistic curve; shaded area, 95% confidence interval) of *Paenibacillus larvae* spore counts in honey (**A**,**B**) and hive debris (**C**) samples. Spore counts were determined by qPCR (**A**,**C**) and plate counting (**B**). The points on the fitted logistic curve show the predicted probability of a colony exhibiting clinical symptoms for each sample.

The univariate logistic regression revealed that plate and qPCR counts in honey samples were associated with the presence of clinical symptoms in the colony (odds ratios: 8.64 and 10.42; confidence intervals: 1.06–4.59 and 1.24–4.45; *p* = 0.0090 and 0.0024, respectively). The same was observed for qPCR counts in hive debris samples (odds ratio: 1.62; confidence interval: 0.29–0.76; *p* < 0.0001). At a probability of 0.5, the threshold to classify colonies as affected (i.e., symptomatic) or unaffected (i.e., asymptomatic) was 1077.21 spores/g honey for qPCR and 6.76 spores/g honey for plate counting (Figure 5). For hive debris, a broader range of qPCR spore counts was observed in the asymptomatic colonies, and a relatively small number of colonies exhibited clinical symptoms, resulting in the logistic regression model not converging (Figure 5). Nevertheless, all the affected colonies harbored >5 log spores/mL debris by qPCR.

For 31/67 honey samples, spore counts were above the LOQ by both methods, enabling calculation of the germination rate (Table S1); it was low and inconsistent (average = 0.52%, range = 0.04–6.05%), making plate counting unsuitable for reliable quantification. Out of 67 honey samples, 66 were positive by qPCR, of which 66 were culture-negative (Table S1), representing a sensitivity of 50/66 (75.8%) for plate counting. All culture-positive samples were also qPCR-positive. All qPCR counts were higher than the plate counts, with an average ratio of qPCR count to plate count of 2.4 logs.

## 4. Discussion

This study is the first report of the use of dPCR for the initial calibration of a novel TaqMan probe-based qPCR assay for the quantification of *P. larvae*. Both previously developed qPCR assays optimized for the detection and quantification of *P. larvae* spores in honey and hive debris [23,26] were based on 16S rRNA gene and SYBR technologies, which are known for their limitations. First, the 16S rRNA gene is present in eight copies per *P. larvae* genome, which can exhibit single nucleotide polymorphisms, resulting in preferential amplification of a particular subset of 16S rRNA gene variants [23]. Second, SYBR-based technology is known to be less specific and sensitive compared with TaqMan-based probes, and highly similar 16S rRNA gene amplicons from closely related species can be difficult to distinguish via melting curve analysis [23,38,39]. The newly developed qPCR assay is based on TaqMan technology and targets a single-copy gene which is highly conserved within the species but absent in other closely related species. The assay overcomes the limitations of previous qPCR assays based on 16S rRNA gene and SYBR chemistry.

Recently, a qPCR assay for the simultaneous detection of *P. larvae* and *M. plutonius* was developed, targeting the *tnp60* gene and *napA* pseudogene, respectively [28]. In the present study, the *tnp60* assay proved unsuitable for the detection and quantification of *P. larvae* because of the variable copy number of the *tnp60* gene, variations between multiple *tnp60* paralogs, and mismatches in the primer/probe binding sites (data not shown).

Here, dPCR was used for initial calibration of qPCR, allowing precise and absolute quantification of *P. larvae* spores in honey and hive debris. Compared with other techniques for the calibration of qPCR, such as plate counting, flow cytometry, and microscopy counting, dPCR represents a *P. larvae*-specific calibration method that circumvents the bias in spore germination. Moreover, it uses the same DNA template, chemistry, and PCR conditions as qPCR, maximizing the comparability of dPCR and qPCR. This was confirmed by the high agreement and strong correlation between qPCR and dPCR observed in this study. Because dPCR is more costly and time-consuming, has a lower sample throughput, and is characterized by a narrower dynamic range compared with qPCR [29], our results suggest that qPCR can effectively replace dPCR for routine detection/quantification of *P. larvae* in honey and hive debris samples. The newly developed qPCR assay is based on widely used TaqMan chemistry and can be extended to other bee-related samples after validation and calibration.

The constructed qPCR assay targeting the metalloproteinase gene showed high performance, with LOD values of 8 spores/g honey and 188 spores/mL hive debris, reaching the theoretical LOD of three targets per reaction [37] both for honey and hive debris. Martínez et al. [26] reported LOD of 2 spores/g of honey, whereas Rossi et al. [23] reported 10 spores/g of honey or hive debris. Both previous assays target the 16S rRNA gene, which is present in eight copies per *P. larvae* genome, and are thus expected to have higher sensitivity compared with the assay developed here. However, as mentioned above, assays based on the 16S rRNA gene and SYBR technology suffer from specificity issues and are less appropriate for reliable quantification. Previous assays employed different methods for qPCR calibration, PCR reagents, and conditions and are, therefore, not directly comparable with the metalloproteinase qPCR assay. In addition, they performed no qPCR replicates [26] or performed only technical replicates [23], thereby not accounting for the inherent biological variability and losses during sample preparation and DNA extraction. An important limitation of the assay developed by Rossi et al. [23], who optimized the assay developed by Martínez et al. [26], is its calibration using plate counting. The metalloproteinase qPCR assay was calibrated using dPCR and validated in accordance with qPCR publication guidelines [37], also considering the inherent variability of *P. larvae* spore counts in complex samples. The assay thus employed validation of the entire process from sample preparation to qPCR.

For honey, the average spore germination rate was 0.52% and showed a broad range (0.04–6.05%), reiterating the previously described discrepancy between molecular and cultivation-based spore counts, which can exceed 1 log unit [22]. The determined germination rate is markedly lower than the previously determined rate of 6%, which was based on the comparison of plate and microscopy counts [40]. Of note, germination rates are also highly dependent on the selection of cultivation/germination media [21,22]; thus, the differences between qPCR and plate counts observed in this study could be decreased if different growth media were to be used. Plate counting had a markedly lower sensitivity (75.8%) than qPCR, whereas none of the culture-positive samples was negative by qPCR, which is in agreement with previous findings [22]. Although qPCR does not enable the distinction between live and dead cells/spores and may lead to an overestimation of qPCR counts, previous work confirmed that two-thirds of culture-negative samples were positive after re-cultivation on modified growth media supplemented with germinant agents [22].

In this study, a significant correlation between the spore counts and the presence/absence of clinical symptoms was observed for both sample types. Spore counts did not differ significantly between the affected colonies with varying levels of disease severity. In the analysis of honey samples that were collected from the brood chamber of individual colonies, a threshold for distinguishing between clinically affected and asymptomatic apiaries at 0.5 probability could be set at ~1000 spores/g honey by qPCR. Spore counts in the hive debris samples collected from asymptomatic colonies varied greatly, making it difficult to establish such a threshold. Of note, all the affected colonies harbored >5 log spores/mL debris by qPCR. The asymptomatic colonies with >5 log spores/mL debris originated from apiaries with histories of AFB (most from apiaries with currently active AFB and fewer from currently AFB-negative apiaries). Gende et al. [14] showed a correlation between higher *P. larvae* spore counts in adult bees from clinically affected colonies compared with asymptomatic colonies and proposed a threshold of ~3000 spores per adult bee for the presence of clinical symptoms in the colony, as determined by plate counting. In this study, spore counts in the asymptomatic colonies also differed significantly with respect to the history of AFB, especially for the hive debris samples. Therefore, the establishment of a threshold (spores per sample unit) to distinguish between affected and unaffected colonies should be considered as a guide rather than a fixed value. Such a threshold is of great importance for future AFB surveillance by qPCR, because it identifies at-risk colonies or apiaries that should undergo clinical examination and isolation of *P. larvae* for final confirmation of AFB.

In Slovenia, there is a very high density of honeybee colonies, on average more than 10 per km^2^ (data from the National Register of Apiaries for 2020, Ministry of Agriculture, Forestry, and Food). Together with the activities of beekeepers (e.g., migratory beekeeping, exchange of equipment, or unreported activities), this promotes the rapid spread of bee diseases. The newly developed qPCR assay provides a cost- and time-efficient means for a country-wide surveillance of AFB. It would enable early detection of at-risk colonies with a high discovery rate. Even if colonies with spore counts close to the threshold are not (yet) showing clinical symptoms, strict control measures should be implemented in such colonies/apiaries (e.g., shaking bees onto new frames, improved hygiene of the beekeeping equipment, strict control of honeybee transfer, and clinical examination of colonies in at-risk apiaries). Early detection and enumeration of *P. larvae* spores provides information on the actual AFB status of honeybee colonies, independent of clinical symptoms; even if they are not yet observed, measures can be activated to prevent the development and spread of this dangerous infectious disease. If the recognition of clinical symptoms is left only to the knowledge and skills of beekeepers who report suspected AFB to the authorities, important time could be wasted for preventing, or at least reducing, the spread of *P. larvae*. When clinical symptoms of AFB are already detected and the presence of *P. larvae* is confirmed, much more effort is required, and great economic losses are observed in the sanitation of the affected apiary and the management of the AFB zones.

In this study, the genotype of *P. larvae* isolates from honey samples was not considered; therefore, the effect of different ERIC types on the disease severity could not be assessed. ERIC types differ in their virulence and influence the disease status [4,31,41]. Thus, future studies on the association between spore counts and disease severity should include *P. larvae* isolates of different ERIC types.

## 5. Conclusions

In summary, we describe here a novel TaqMan-based qPCR assay for reliable quantification of *P. larvae* in bee-related samples (honey and hive debris) which was calibrated using dPCR. A significant correlation was found between increased qPCR counts and the presence of AFB clinical symptoms, both for honey and hive debris samples. The culture-based method, which is routinely used for AFB surveillance, was shown to be unreliable for detection/quantification of *P. larvae*, due to poor and inconsistent spore germination. The metalloproteinase qPCR assay allows a more sensitive, rapid, and specific detection/quantification of *P. larvae* and will lead to improved surveillance of AFB.

## Figures and Tables

**Table 1:** dPCR quantification of the DNA dilution series prepared from positive honey (H) and hive debris (D) samples used for calibration of qPCR. dPCR counts for the negative control samples (NTC for honey, NTC for hive debris, and WNTC), collected from different dPCR runs, are also given to show the differences in precision and the maximum number of positive wells per chip. The average numbers were calculated from at least four technical dPCR replicates (chips). See Section 2.6 for details.

dPCR	Spores/µL DNA (Range) *	Precision [%]	Avg. No. of Positive Wells/Chip ** (% of All Filled)	Avg. No. of Negative Wells/Chip ** (% of All Filled)
Positive (H)	27,125 (24,767–28,587)	1.3	–	–
Positive (D)	243,000 (154,000–293,000)	0.9	–	–
NTC (H)	1.2 (0.4–2.8)	229.9	3.0 (0.2)	17,149 (94.5)
NTC (D)	0.6 (0.2–1.9)	104.2	3.5 (0.2)	16,971 (94.2)
WNTC	1.0 (0.2–1.6)	137.9	4.2 (0.2)	17,348 (95.1)

NTC, negative template control; WNTC, water no template control. * DNA dilution rate was considered to calculate the number of spores/µL of the extracted DNA in the positive calibration samples. For each technical replicate of the biological replicates, more than one dilution was quantified by dPCR. “Spores” refers to spore equivalents as determined by dPCR, since the assay targets a single-copy metalloproteinase gene. For the negative control samples, the calculated number of spores is a result of false-positive dPCR events, since the samples contained no *P. larvae* spores determined by qPCR or plate counting and precision was far outside the acceptable range to obtain reliable results. ** For the positive calibration samples, the number of positive or negative wells/chip is not reported, since it depends on the analyzed DNA dilution. On average, the number of wells/chip filled (18,517 with a range of 18,107–18,834 for honey; 18,528 with a range of 18,061–19,173 for hive debris) and qualified by quality threshold (17,513 with a range of 16,818–17,888 for honey; 16,935 with a range of 16,164–17,609 for hive debris) was comparable with those of the negative control samples (18,153 filled, with range 17,447–18,771, and 17,187 qualified, with range 16,263–18,125), depending on the applied dPCR technology.

## Data Availability

Experimental dataset supporting the reported results is provided in Tables S1 and S5.

## Supplementary Materials

The following are available online at https://www.mdpi.com/article/10.3390/insects12111034/s1: Figure S1. *Paenibacillus larvae* metalloproteinase primer/probe binding sites. Table S1. Association between AFB clinical symptoms and *Paenibacillus larvae* counts determined by qPCR and plate counting for honey and hive debris samples. Table S2. Scale for grading the severity of AFB clinical symptoms. Table S3. In silico qPCR inclusivity and exclusivity. Table S4. In vitro qPCR inclusivity and exclusivity. Table S5. Comparison of dPCR and qPCR for honey and hive debris samples.
