# Supplementary material for: Novel TaqMan PCR Assay for the Quantification of Paenibacillus larvae Spores in Bee-Related Samples
Source: Insects. 2021 Nov 17;12(11):1034. doi: 10.3390/insects12111034 (PMC8621733; doi:10.3390/insects12111034)
Supplement: Supplementary file 1 [file insects-12-01034-s001.zip › insects-1458549-supplementary/Figure S1.pdf]

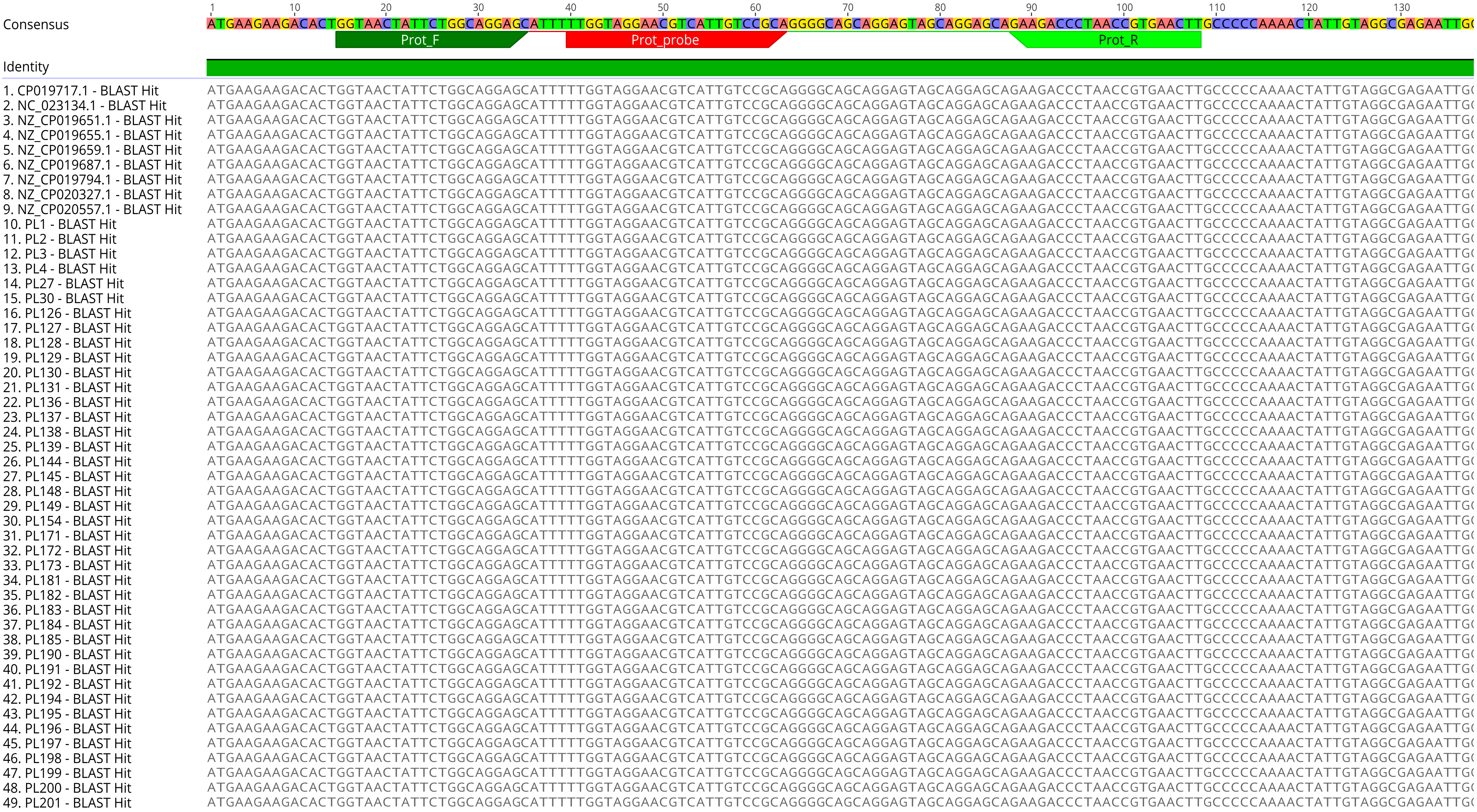


**Figure S1.** *Paenibacillus larvae* metalloproteinase primer/probe binding sites. Alignment of *P. larvae* metalloproteinase gene sequences was constructed from sequences of nine complete genomes and 40 draft genome sequences of field strains. For isolate metadata, see Table S3.
