# Supplementary material for: Novel TaqMan PCR Assay for the Quantification of Paenibacillus larvae Spores in Bee-Related Samples
Source: Insects. 2021 Nov 17;12(11):1034. doi: 10.3390/insects12111034 (PMC8621733; doi:10.3390/insects12111034)
Supplement: Supplementary file 1 [file insects-12-01034-s001.zip › insects-1458549-supplementary/Table S2.pdf]

Table S2. Scale for grading the severity of AFB clinical symptoms.

Scale	Clinical symptoms
**0**	No AFB clinical symptoms
**1**	Low level of scattered/patchy bee brood<br>Low number (1–2 cells per comb) of cell cappings are concave, darkened and/or wet-looking)<br>Some ropy larvae/cell mass of milky to light brown coloration<br>Clinical symptoms are limited to a single comb per colony<br>Colony strength is not affected
**2**	Scattered/patchy bee brood is clearly visible<br>Bee larvae on individual combs vary in age<br>Some cell cappings are concave, darkened and/or wet-looking<br>Up to five perforated cell cappings per comb<br>Ropy larvae/cell mass of light to dark brown coloration<br>Up to three affected combs per colony<br>Colony strength is slightly decreased
**3**	High level of scattered/patchy bee brood<br>Bee larvae on individual combs vary in age<br>A lot of cell cappings are concave, darkened and/or wet-looking<br>More than five perforated cell cappings per comb<br>Ropy larvae/cell mass of light to dark brown coloration<br>Clinical symptoms are present in more than three combs per colony<br>Colony strength is considerably decreased
**4***	Only few remaining capped cells on combs<br>Most cell cappings are perforated<br>Ropy larvae/cell mass of dark brown coloration<br>Dried-out larvae form a hard scale, that adheres to the cell wall (tongue appearance)<br>Colony strength is highly decreased or there are only a few remaining bees in the hive<br>Colony is robbed

*One honeybee colony was scaled '4' and was interpreted here as '3' for the purpose of statistical analysis.
